# What is the impact of ongoing statin use on cognitive decline and dementia progression in older adults with mild-moderate Alzheimer disease?

**DOI:** 10.1371/journal.pone.0285529

**Published:** 2023-05-11

**Authors:** Claire Murphy, Adam H. Dyer, Brian Lawlor, Sean P. Kennelly, for the NILVAD study group

**Affiliations:** 1 Department of Age-Related Healthcare, Tallaght University Hospital, Dublin, Ireland; 2 Department of Medical Gerontology, School of Medicine, Trinity College Dublin, Dublin, Ireland; 3 Global Brain Health Institute, Trinity College Dublin, Dublin, Ireland; Instituto Nacional de Geriatria, MEXICO

## Abstract

**Background:**

In addition to the clear cardiovascular benefit, there has been renewed interest in the potential of statins in the prevention of cognitive impairment and dementia in older adults. However, whether ongoing statin use can delay cognitive decline or dementia progression in those with established Alzheimer dementia, is unclear.

**Methods:**

Using data from NILVAD, we analysed the association between ongoing statin use and cognitive decline (Alzheimer Disease Assessment Scale-Cognitive Subsection [ADAS-Cog])/dementia progression (Clinical Dementia Rating Scale [CDR-Sb]/Disability Assessment for Dementia [DAD]) over 18 months in older adults with mild-moderate AD. Additionally, we assessed the association between ongoing statin use and adverse events in mild-moderate AD.

**Results:**

Over one-third (34.9%) of 510 older adults with mild-moderate AD (aged: 72.9 years; 61.9% female) used a statin for the 18-month study duration. Statin use was not associated with the rate of cognitive decline (β: -0.67; 95% CI: -1.71, 0.36, p = 0.20) or dementia progression (β: -0.34; 95% CI -0.71, 0.02; p = 0.07 for CDR-Sb/ β: -2.00; -5.70, 1.70; p = 0.29 for DAD). Further, ongoing statin use was not associated with adverse events, serious adverse events, unscheduled GP visits, or unscheduled hospitalisation.

**Conclusion:**

Ongoing statin use was not associated with cognitive decline or dementia progression in mild-moderate AD. Similarly, use was not associated with adverse events including abnormal liver function tests or falls. Whilst safe in those with AD, the current results suggest ongoing statin use does not delay cognitive decline or clinical progression in established AD.

## Introduction

The prescribing of statins for both primary and secondary prevention of atherosclerotic cardiovascular disease is increasing [1]. In older adults, the use of statins for secondary prevention is well-established, with statin use associated with reduced incidence of cardiovascular disease, cardiovascular death, fatal and non-fatal stroke [2–4]. Their use in people with dementia is less well studied. The co-existence of neurodegenerative disease and cardiovascular disease is common in older adults. Alzheimer Dementia (AD) and vascular disease dementia are the two most common types of dementia. Increasingly, AD and vascular pathology have been recognised to co-occur often as mixed pathology in cases of dementia [5]. This highlights the potential role for statins in the prevention and treatment in people living with AD however there is no consensus on the use of statins in this group. In fact a recent systematic review [6] demonstrated much of the research to date on statin use in older people excludes those with a diagnosis of dementia.

The existing literature surrounding statin use in people living with AD is conflicting both their role in prevention but also their use in those with established cognitive impairment or Dementia [7–10]. Many retrospective observational studies have demonstrated a reduced incidence of dementia in statin users [8–11] with some studies specifically demonstrating a protective effect of statins in the development of AD [12]. However this was not confirmed in randomised controlled trials and an early Cochrane review in 2001 [13] and subsequent reviews [14] failed to demonstrate a protective role of statins in the development of dementia. The effect of statin use on the cognition in those with established disease is less well studied but also debated. Some studies have suggested improved cognitive scores in statin users with others including a recent Cochrane review demonstrating no effect [15–17].

In addition to the inconsistencies in the evidence for their use in the prevention and treatment of dementia, more recently concerns over reversible cognitive impairment in statin users have resulted in the FDA issuing a black-box warning to healthcare professionals against reversible statin related cognitive impairment [18]. This evidence is primarily based on case reports and early randomised control trials [19] in people without an existing diagnosis of dementia.

In the current study, we assessed the use of statins in an international population with established mild-to-moderate AD to analyse the effect of statin use on cognitive performance. We also assessed adverse outcomes such as unscheduled hospitalisations, unscheduled attendances at the participants regular General Practitioner (GP) and more specifically liver function tests and falls both previously reported side effects of statin use [20,21].

## Methods

### Study background and setting

The current study utilised data from NILVAD (Clinicaltrials.gov NCT02017340; EudraCT number 2012-002764-27). NILVAD was a phase-three international randomised control clinical trial investigating the effect of *Nilvadipine* on patients with mild to moderate Alzheimer’s Disease (AD). This study was negative for its primary end point of change in cognitive decline/dementia severity at 18 months. Participants were recruited from 23 academic centres across nine European countries (Ireland, United Kingdom, Italy, the Netherlands, France, Greece, Sweden, Germany and Hungary). All participants had a diagnosis of mild-moderate AD defined using the National Institute of Neurological and Communicative Disorders and Stroke/Alzheimer’s disease Criteria (NINCDS-ADRDA) with a Standardised Mini-Mental State Examination (sMMSE) score from 12–26. Participants included were 50 years or older. Further details of inclusion / exclusion criteria can be found in the original study protocol [22,23]. Ethical approval was granted from appropriate National Competent Authorities, Independent Ethics Committees and Institutional Review Boards for all study 25 sites.

### Cognitive and dementia severity assessment

Cognitive scores were assessed at baseline and at three follow up visits (13, 52 and 78 weeks). The Alzheimer Disease Assessment Scale, Cognitive Subsection (ADAS-Cog) was used to evaluate cognitive impairment whilst the Clinical Dementia Rating Scale-Sum of Boxes (CDR-sb) and Disability Assessment for Dementia (DAD) were used to assess AD severity.

### Medication records, medical history and assessment

Medication records and co-morbidities were available for included participants across all centres in addition to information on relevant cardiovascular risk factors including age, sex, diabetes, blood pressure and body mass index. Medications were coded using the Anatomic Therapeutic Classification (ATC) System and co-morbidities coded using the International Classification of Diseases (ICD-10) System. Polypharmacy was defined as being prescribed five or more medications. Diabetes Mellitus was defined as a self-reported history of same or being prescribed medications only listed for the treatment of Diabetes Mellitus. Blood Pressure, recorded in the seated position after five minutes rest, was divided into the following categories: (i) high: ≥ 140/90 mmHg (ii) normal: 130–139 / 70–89 mmHg and (iii) low: <130/70 mmHg. Fasting blood tests were performed locally at each study site at both baseline and study close-off. Liver Function Tests (LFTs) included: albumin, total protein, alanine aminotransferase, aspartate aminotransferase, alkaline phosphatase and gamma-glutamyl transferase. Abnormal LFTs were defined as any abnormal LFT at either 0 or 18 months.

Statin use was defined as use of any agent with the ATC code “C10AA” (HMG-CoA Reductase Inhibitors). Statin users were those using Statins for the entire 18 month study duration. Non Statin users were those not using any statin for the 18 month study duration.

### Reporting of Averse events

At each study visit during the NILVAD trial participants (and caregivers as appropriate) were asked about recent adverse events or serious adverse events, this included any new symptoms or clinical incidents and specific questions regarding cognitive status and delirium. Participants were also asked re unscheduled GP visits and unscheduled hospital visits. This information was collected at weeks 0, 6, 13, 26, 39, 52, 65 and 78.

### Statistical analysis

STATA V.15 (Stata Corp, College Station, Texas, USA) was used for all data analysis in the study with p<0.05 considered statistically significant. Between group univariate analysis was conducted using T Tests, Wilcoxon rank sum tests and chi-squared tests as appropriate. Descriptive statistics are reported as means (standard deviations), medians (interquartile ranges) and proportions (percentages).

In order to analyse the association of ongoing statin use on change in ADAS-Cog/CDR-Sb/DAD over 18 months, mixed effects linear regression was used, with study site as a random effect. The association between statin use and the dependent variable was modelled using a statin*time interaction term. This was tested unadjusted (Model 1) in the first instance, followed by adjustment for age, gender, baseline score (ADAS-Cog/CDR-Sb/DAD as appropriate), study group (Nilvadipine vs placebo) and years of formal education (Model 2). Model 3 made further adjustment for blood pressure, diabetes mellitus and polypharmacy.

In order to examine the association between ongoing statin use and incident adverse events/serious adverse events over the study duration, Poisson regression was used, again unadjusted in the first instance (Model 1) followed by further adjustment for age, gender body mass index, education and study group (Model 2). In model 3, adjustment was made for baseline ADAS-Cog and CDR-Sb in addition to medical co-morbidity (no. of medical co-morbidities) and polypharmacy. To assess potential associations between statin use and deranged Liver Function Tests (LFTs) and incident falls, we used logistic regression with the same model adjustment as the Poisson models.

## Results

### Participant characteristics

Of 510 participants included in the NILVAD Study (mean age: 72.9 years; 61.9% female), 34.9% (178/510) were prescribed a statin for the 18 month study duration. Statin users vs non-users did not differ significantly in age, gender, blood pressure category (as outlined above), educational attainment, history of diabetes and BMI. There was a significantly greater burden of polypharmacy and medical co-morbidity in statin users vs. non-users (χ^2^ = 40.2, p<0.001, z = -6.4, p<0.001 respectively). See Table 1 for detailed baseline and demographic characteristics.

**Table 1:** Baseline characteristics of participants by statin use.

Baseline Characteristic	No Statin (N = 332)	Statin (N = 178)	Statistic
Age, mean years (SD)	72.6 (8.6)	73.5 (7.6)	t = 1.24, p = 0.11
Gender, N (%) female	196 (59.2%)	119 (66.9%)	χ^2^ = 2.86, p = 0.09
Group, N (%) Nilvadipine	170 (51.4%)	87 (48.9%)	χ^2^ = 0.29, p = 0.59
Cholinesterase Inhibitor, N (%)	289 (87.3%)	161 (90.5%)	χ^2^ = 1.11, p = 0.29
Education, mean (SD)	16.7 (4.4)	15.9 (3.7)	t = 2.26, p = 0.01*
Polypharmacy (> 5 medications), N(%)	100 (30.1%)	105 (59.0)	χ^2^ = 40.2, p<0.001**
Number of Medical Comorbidities, median (IQR)	3 (2–5)	4 (3–6)	z = -6.4, p<0.001**
Years since Diagnosis, mean years (SD)	1.6 (0.09)	1.8 (1.9)	t = 1.05, p = 0.15
Body Mass Index (BMI), mean mg/kg^2^ (SD)	25.2 (4.3)	25.9	t = 1.92, p = 0.03*
Diabetes Mellitus	17 (5.69%)	20 (11.9%)	χ^2^ = 5.70, p = 0.02*
BP Category *Low* *Normal* *High*	57 (18.4%) 132 (42.6%) 121 (39.0%)	33 (20.8%) 60 (37.7%) 66 (41.5%)	χ^2^ = 1.07, p = 0.59
Baseline ADAS-Cog, mean (SD)	34.9 (10.9)	33.8 (9.9)	t = 1.13, p = 0.13
Baseline CDR-Sb, mean (SD)	5.5 (2.7)	4.9 (2.8)	t = 2.21, p = 0.01*
Baseline DAD, mean (SD)	28.7 (7.9)	29.9 (7.9)	t = 1.77, p. = 0.04*

SD = Standard Deviation

N = Number

BMI = Body Mass Index

ADAS-Cog = Alzheimer’s Disease Assessment Scale Cognitive Subsection

CDR-Sb: Clinical Dementia Rating Score

Sum of Boxes

DAD: Disability Assessment for Dementia.

### Is ongoing statin use associated with cognitive decline and dementia severity?

On longitudinal analysis, there was no association between ongoing statin use and the rate of either cognitive decline (ADAS-Cog) under any of the three models specified above (See Table 2). A trend for a slower rate of dementia progression was observed on the CDR-Sb with statin use, although results were not statistically significant (β: -0.34, -0.71–0.02, p = 0.07 unadjusted; -0.37, -0.74–0.01, p = 0.05 for model 1; -0.37, -0.76–0.02, p = 0.06 for model 2). Similarly, there was no association between ongoing statin use and change in scores on the DAD.

**Table 2:** Statin use, cognitive decline and dementia progression over 18 months.

	Model 1		Model 2		Model 3	
Predictor	*β* -Coef (95% CI)	*P Value*	*β* -Coef (95% CI)	*P Value*	*β* Coef (95% CI)	*p Value*
***Change in ADAS-Cog***						
Statin Use* Visit	-0.67 (-1.71, 0.36)	0.20	-0.74 (-1.77, 0.29)	0.16	-0.77 (-1.86, 0.33)	0.17
Age			-0.12 (-0.17, -0.07)	<0.001**	-0.14 (-0.19, -0.08)	<0.001*
Gender (Female)			-0.34 (-1.19, 0.51)	0.43	-0.60 (-1.51, 0.32)	0.20
Baseline ADAS-Cog			0.05 (0.00, 0.09)	0.03	0.04 (-0.01, 0.08)	0.09
Group (Nilvadipine)			0.14 (-0.67, 0.95)	0.73	-0.09 (-0.98, 0.80)	0.83
BMI, mg/kg^2^			-0.17 (-0.26, -0.07)	0.001*	-0.17 (-0.27, -0.07)	0.001*
Years of Formal Education			0.02 (-0.09, 0.13)	0.68	0.07 (-0.05, 0.19)	0.26
Blood Pressure (Category) *Low* *Normal* *High*					1. (Ref) 0.61 (-0.63, 1.85) 0.09 (-1.16, 1.35)	0.34 0.88
Diabetes Mellitus					0.57 (-2.27, 1.13)	0.51
Total Comorbidities					0.13 (-0.07, 0.33)	0.19
Polypharmacy					0.90 (-0.12, 1.91)	0.08
***Change in CDR-Sb***						
Statin Use* Visit	-0.34 (-0.71, 0.02)	0.07	-0.37 (-0.74, 0.01)	0.05	-0.37 (-0.76, 0.02)	0.06
Age			-0.03 (-0.05, -0.01)	0.002*	-0.03 (-0.05, -0.01)	0.001*
Gender (Female)			-0.16 (0.46, -0.14)	0.29	-0.19 (-0.51, 0.13)	0.25
Baseline CDR-Sb			0.10 (0.05, 0.16)	<0.001*	0.09 (-0.03, 0.15)	0.003*
Group (Nilvadipine)			0.06 (-0.23, 0.35)	0.70	-0.04 (0.35, 0.29)	0.82
BMI, mg/kg^2^			-0.01 (-0.05, 0.02)	0.47	-0.01 (-0.05, 0.02)	0.47
Years of Formal Education			-0.02 (-0.06, 0.02)	0.83	-0.01 (-0.05, 0.04)	0.81
Blood Pressure (Category) *Low* *Normal* *High*					1. (Ref) 0.26 (-0.18, 0.70) 0.15 (-0.29, 0.59)	0.24 0.51
Diabetes Mellitus					0.12 (-0.49, 0.72)	0.72
Total Comorbidities					-0.01 (-0.07, 0.07)	0.71
Polypharmacy					0.48 (0.12, 0.83)	0.01*
***Change in DAD***						
Statin Use* Visit	-2.00 (-5.70, 1.70)	0.29	-1.91 (-5.65, 1.82)	0.31	-2.44 (-6.57, 1.69)	0.25
Age			0.22 (0.04, 0.41)	0.02*	0.26 (0.05, 0.48)	0.02*
Gender (Female)			0.81 (-2.26, 3.89)	0.60	1.45 (-1.98, 4.88)	0.41
Baseline DAD			-0.04 (-0.23, 0.15)	0.71	-0.06 (0.27, 0.16)	0.61
Group (Nilvadipine)			-0.78 (-3.73, 2.16)	0.60	-1.06 (-4.41, 2.30)	0.54
BMI, mg/kg^2^			-0.21 (-0.55, 0.14)	0.24	-0.21 (-0.60, 0.17)	0.28
Years of Formal Education			0.16 (-0.22, 0.54)	0.40	0.23 (-0.21, 0.67)	0.31
Blood Pressure (Category) *Low* *Normal* *High*					1. (Ref) 1.32 (-3.33, 5.96) 0.39 (-4.34, 5.12)	0.58 0.87
Diabetes Mellitus					-3.47 (-9.87, 2.93)	0.29
Total Comorbidities					0.06 (-0.67, 0.79)	0.87
Polypharmacy					-0.51 (-4.33, 3.31)	0.79

BMI = Body Mass Index

ADAS-Cog = Alzheimer’s Disease Assessment Scale Cognitive Subsection

CDR-Sb: Clinical Dementia Rating Score

Sum of Boxes

DAD: Disability Assessment for Dementia.

### Is ongoing statin use associated with incident adverse events?

Overall, statin users reported a median of 3 (IQR: 1–6) adverse events as did non-users (median 3: IQR: 1–6). A similar number in both groups experienced a serious adverse event (61/333; 18.3% for non-users vs 30/178; 16.9% for statin users). Ongoing statin use was not associated with incident adverse events or serious adverse events under either unadjusted or fully adjusted models. Further, there was no association between ongoing statin use and incident unscheduled GP visits or unscheduled hospitalisations. See Table 3. Overall, 181 (37.9%) participants had at least one abnormal LFT at either 0 or 18 months. There was no association between statin use and abnormal LFTs. Further, whilst 88 individuals (17.9%) experienced at least one fall over the study period, there was no significant association between statin use and incident falls. See Table 4.

**Table 3:** Statin use and association with adverse events, serious adverse events, unscheduled hospitalisations and unscheduled GP visits over 18 months.

	IRR (95% CI)	P Value	IRR (95% CI)	P Value	IRR (95% CI)	P Value
***Adverse Event***						
Statin Use	1.09 (0.99, 1.18)	0.07	1.09 (0.99, 1.18)	0.08	0.93 (0.85, 1.02)	0.13
Age, years			1.00 (1.00, 1.01)	0.004*	1.00 (0.99, 1.01)	0.35
Gender (Female)			1.14 (1.04, 1.25)	0.05	1.10 (1.00, 1.20)	0.04*
Group (Nilvadipine)			0.92 (0.84, 1.01)	0.08	0.92 (0.85, 1.00)	0.07
Education, years			1.03 (1.02, 1.04)	<0.001**	1.03 (1.02, 1.05)	<0.001**
BMI, mg/kg^2^			0.99 (0.98, 1.00)	0.16	0.99 (0.98, 1.00)	0.03*
Baseline ADAS-Cog					1.00 (0.99, 1.01)	0.76
Baseline CDR-Sb					1.00 (0.97, 1.02)	0.64
Total No. Comorbidities					1.03 (1.01, 1.05)	0.004*
Polypharmacy					1.58 (1.43, 1.73)	<0.001**
***Serious Adverse Event***						
Statin Use	0.93 (0.70, 1.22)	0.57	1.02 (0.77, 1.35)	0.90	0.77 (0.57, 1.03)	0.08
Age, years			1.05 (1.03, 1.08)	<0.001	1.04 (1.03, 1.06)	<0.001
Gender (Female)			0.82 (0.62, 1.07)	0.15	0.73 (0.55, 0.97)	0.03
Group (Nilvadipine)			0.67 (0.50, 0.87)	0.003	0.66 (0.51, 0.87)	0.003
Education, years			1.05 (1.02, 1.08)	0.001	1.07 (1.04, 1.10)	<0.001
BMI, mg/kg^2^			0.96 (0.93, 0.99)	0.03	0.96 (0.93, 0.99)	0.01
Baseline ADAS-Cog					1.00 (0.98, 1.02)	0.97
Baseline CDR-Sb					1.07 (1.00, 1.14)	0.05
Total No. Comorbidities					1.04 (0.99, 1.09)	0.15
Polypharmacy					2.62 (1.93, 3.55)	<0.001
***Unscheduled GP Visits***						
Statin Use	1.33 (1.08, 1.64)	0.008	1.28 (1.04, 1.59)	002	1.01 (0.80, 1.27)	0.93
Age, years			1.00 (0.99, 1.02)	0.58	1.00 (0.98, 1.01)	0.62
Gender (Female)			1.12 (0.90, 1.40)	0.32	1.05 (0.84, 1.32)	0.64
Group (Nilvadipine)			0.94 (0.76, 1.15)	0.53	0.94 (0.76, 1.16)	0.56
Education, years			1.01 (0.99, 1.04)	0.32	1.02 (0.00, 1.05)	0.08
BMI, mg/kg^2^			1.02 (0.99, 1.04)	0.12	1.01 (0.99, 1.04)	0.32
Baseline ADAS-Cog					1.01 (0.99, 1.02)	0.17
Baseline CDR-Sb					0.98 (0.93, 1.03)	0.47
Total No. Comorbidities					1.05 (1.01, 1.10)	0.04
Polypharmacy					1.98 (1.56, 2.51)	<0.001
***Unscheduled Hospitalisations***						
Statin Use	0.91 (0.59, 1.41)	0.67	0.93 (0.60, 1.46)	0.77	0.81 (0.51, 1.29)	0.37
Age, years			1.03 (1.00, 1.06)	0.03	1.02 (0.99, 1.05)	0.12
Gender (Female)			1.05 (0.68, 1.61)	0.84	1.00 (0.65, 1.55)	0.99
Group (Nilvadipine)			0.78 (0.52, 1.19)	0.25	0.80 (0.53, 1.21)	0.29
Education, years			1.04 (0.99, 1.09)	0.16	1.04 (0.99, 1.10)	0.10
BMI, mg/kg^2^			0.99 (0.94, 1.04)	0.57	0.98 (0.93, 1.03)	0.46
Baseline ADAS-Cog					0.99 (0.96, 1.02)	0.56
Baseline CDR-Sb					1.06 (0.96, 1.18)	0.26
Total No. Comorbidities					1.03 (0.95, 1.13)	0.44
Polypharmacy					1.64 (1.04, 2.58)	0.04

BMI = Body Mass Index

ADAS-Cog = Alzheimer’s Disease Assessment Scale Cognitive Subsection

CDR-Sb: Clinical Dementia Rating Score, Sum of Boxes.

**Table 4:** Statin use was not associated with abnormal LFTs or incident falls.

	OR (95% CI)	P Value	OR (95% CI)	P Value	OR (95% CI)	P Value
***Abnormal LFTs***						
Statin Use	1.11 (0.76, 1.63)	0.59	1.14 (0.77, 1.68)	0.53	1.09 (0.72, 1.66)	0.69
Age, years			0.98 (0.96, 1.00)	0.12	0.97 (0.95, 1.00)	0.05
Gender (Female)			0.85 (0.57, 1.25)	0.40	0.85 (0.57, 1.26)	0.41
Group (Nilvadipine)			1.03 (0.71, 1.49)	0.90	1.06 (0.73, 1.54)	0.77
Education, years			1.00 (0.96, 1.05)	0.95	1.00 (0.96, 1.05)	0.87
BMI, mg/kg^2^			1.02 (0.98, 1.07)	0.40	1.02 (0.97, 1.06)	0.51
Baseline ADAS-Cog					0.98 (0.96, 1.01)	0.22
Baseline CDR-Sb					1.10 (1.00, 1.21)	0.06
Total No. Comorbidities					1.02 (0.94, 1.12)	0.59
Polypharmacy					1.20 (0.79, 1.82)	0.41
***Falls***						
Statin Use	0.***90*** (0.55, 1.47)	0.67	0.89 (0.53, 1.49)	0.67	0.80 (0.46, 1.38)	0.42
Age, years			1.06 (1.03, 1.09)	<0.001	0.80 (0.46, 1.38)	<0.001
Gender (Female)			2.66 (1.51, 4.66)	<0.001	2.58 (1.47,4.56)	<0.001
Group (Nilvadipine)			0.94 (0.58, 1.52)	0.80	0.96 (0.59, 1.56)	0.86
Education, years			1.08 (1.02, 1.15)	0.01	1.09 (1.03, 1.16)	0.01
BMI, mg/kg^2^			0.94 (0.88, 0.99)	0.04	0.94 (0.88, 0.99)	0.03
Baseline ADAS-Cog					0.99 (0.96, 1.03)	0.78
Baseline CDR-Sb					1.03 (0.91, 1.03)	0.65
Total No. Comorbidities					1.00 (0.89, 1.11)	0.99
Polypharmacy					1.63 (0.95, 2.78)	0.07

LFTs = Liver function tests

BMI = Body Mass Index

ADAS-Cog = Alzheimer’s Disease Assessment Scale Cognitive Subsection

CDR-Sb: Clinical Dementia Rating Score, Sum of Boxes.

## Discussion

The current study demonstrates that ongoing use of statins was not associated with cognitive decline or worsening of dementia severity in community-dwelling older adults with mild-moderate AD. Further, use of these medications was not associated with an increased risk of adverse events, serious adverse events or unscheduled healthcare utilisation. Our findings are reassuring for both the effect on cognitive function and safety profile of statins in older adults with AD.

As previously outlined the existing literature on the effect of statins in this group has been conflicting. A large scale RCT by Feldman et al [15] investigating the role of atorvastatin in mild to moderate Alzheimer’s disease demonstrated no change in cognitive scores (ADAS-cog) or function (ADCS-CGIC). More recently Xuan et al [16] demonstrated in a systematic review and meta-analysis of nine randomised control trials a potential short term improvement in mini mental state examination (MMSE), Neuropsychiatric inventory (NPI) and activities of daily living scale (ADL) with the use of statins in people with established AD but no change in Alzheimer’s disease assessment scale–cognitive (ADAS-Cog). Our study is one of the largest to date to assess statin use and the effect on cognition in participants with established AD. This study is strengthened by the scope of the data available on individual participants allowing control for multiple relevant covariates. The extensive cognitive testing which participants underwent with three validated cognitive assessment tools used adds to the validity of this study and the conclusions that statin use was not associated with cognitive decline in this cohort.

The well recognised adverse effects of statins including myopathy, myositis, gastrointestinal upset, deranged LFTs and Diabetes are exposure dependent with an increased risk for longer duration and at higher doses. There is an increased risk of adverse events with increasing co morbidity and polypharmacy increasing the risk of drug—drug interactions. Older people with dementia have higher incidence of multi morbidity and polypharmacy than those without dementia [24]. The safety profile in older people is well established [25] with some supportive evidence in those with established AD but again this group is less well studied, our study including this cohort.

The inclusion of international participants with multi-morbidity and polypharmacy adds to the generalisability of this study. This is particularly pertinent when analysing the potential adverse events of statins in this cohort. Older patients with multi-morbidity, defined as two or more concurrent chronic medical conditions are at increased risk of drug-drug interactions, adverse drug events and serious adverse drug events [26,27]. This study demonstrates no increase in adverse drug events or serious adverse drug events associated with stain use in this population. Polypharmacy, multi morbidity and dementia are associated with increased frequency of hospitalisation and prolonged hospital stays [28,29]. This study demonstrates no increase in unscheduled hospitalisations or unscheduled GP visits associated with the use of statins. Whilst we appreciate that GP and hospital visits are critical to the overall care of people with dementia avoidance of unscheduled care is desirable.

Whilst our findings demonstrated no effect of statin use in the rate of cognitive decline or dementia progression in mild-moderate AD, it is possible that statin use in earlier years (for instance in midlife) may have beneficial cognitive effects not observed here but reported elsewhere [30,31]. There may also be a role in those with early mild cognitive impairment as suggested by Kemp et al [32] which warrants further study. Our use of a cohort with established cognitive impairment is noteworthy given the lack of studies addressing this population, but it must be noted that we cannot deduce conclusions on the role of statins in dementia prevention in the first instance, only on the rate of decline in those with established disease. As this is a retrospective observational analysis and statins were initiated prior to enrolment in the trial we did not have data on individual participants serum cholesterol pre or post statin use, this may be an area for future consideration.

## Conclusion

The findings of this study support the continued use of statins in their role to reduce the risk of atherosclerotic cardiovascular disease in people with dementia. There was no adverse effect on cognition associated with their use demonstrated. The use of statins has been shown to be safe with no increased risk of adverse events and we would recommend their use where indicated for cardiovascular health in people with mild to moderate Alzheimer’s disease.
